# Relationship Between Cardiac Troponin I Concentration and Myocardial Function in Hypertrophic Cardiomyopathy Cats With or Without Left Ventricular Outflow Tract Obstruction

**DOI:** 10.3390/ani15091313

**Published:** 2025-05-01

**Authors:** Shuji Satomi, Ryohei Suzuki, Yunosuke Yuchi, Yayoi Yoshii, Haruka Kanno, Takahiro Teshima, Hirotaka Matsumoto

**Affiliations:** 1 Laboratory of Veterinary Internal Medicine, School of Veterinary Medicine, Faculty of Veterinary Science, Nippon Veterinary and Life Science University, Tokyo 180-8602, Japan; jetlog21117@yahoo.co.jp (S.S.); y.0301.yunosuke@gmail.com (Y.Y.); teshima63@nvlu.ac.jp (T.T.); matsumoto@nvlu.ac.jp (H.M.); 2 Pet Clinic Lusty, Osaka 545-0011, Japan; 3 Garden Veterinary Hospital, Tokyo 153-0063, Japan

**Keywords:** cardiac biomarker, echocardiography, feline, heart, left ventricular outflow obstruction, myocardial function, myocardial injury, speckle tracking, strain, systolic anterior motion

## Abstract

Hypertrophic cardiomyopathy (HCM) is the most common cardiac disease in cats. HCM is classified into obstructive HCM (HOCM) and non-obstructive HCM (HNCM) forms based on the presence or absence of left ventricular outflow tract obstruction (LVOTO). In humans, LVOTO is a known poor prognostic factor and a target for therapeutic intervention. In contrast, LVOTO has been reported not to contribute to prognosis in cats. This study demonstrated significant correlations between cTnI levels and a maximal LV outflow tract velocity (LVOTV). This finding suggests that LVOTO is associated with myocardial cell injury.

## Simple Summary

Hypertrophic cardiomyopathy is the most common cardiac disease in cats. In some cases, left ventricular outflow tract obstruction is observed, which is considered a poor prognostic factor in humans. In contrast, left ventricular outflow tract obstruction in cats is not regarded as a prognostic factor, since its pathophysiology remains poorly understood. This study aimed to evaluate myocardial injury and function in cats with hypertrophic cardiomyopathy based on the presence of left ventricular outflow tract obstruction. The results demonstrated a close association between left ventricular outflow tract obstruction and myocardial injury.

## 1. Introduction

Hypertrophic cardiomyopathy (HCM) is the most common cardiac disease in cats and is characterized by myocardial concentric hypertrophy [1,2]. In some cases, HCM results in left ventricular (LV) outflow tract obstruction (LVOTO) due to systolic anterior motion of the mitral valve (SAM). Based on the presence or absence of LVOTO, it is classified as obstructive HCM (HOCM) or non-obstructive HCM (HNCM).

Recently, 2D speckle-tracking echocardiography (2D-STE) has been utilized for diagnosing and evaluating myocardial dysfunction in both humans and cats with HCM [3,4,5,6,7,8]. Specifically, because myocardial strain obtained from 2D-STE could evaluate multidirectional myocardial function both locally and comprehensively, 2D-STE would be expected to provide more detailed myocardial function than conventional functional indices [9]. Moreover, a previous feline study has reported worsened myocardial function in cats with HOCM than in cats with HNCM [3].

Cardiac troponin I (cTnI) is a cardiac biomarker that reflects myocardial injury [10,11,12]. Recently, in humans, it has been reported that alleviation of LVOTO through medical management leads to a reduction in cTnI levels [13]. Additionally, the administration of anti-myosin drugs to patients with HOCM led to an improvement in LVOTO, accompanied by a decrease in cTnI levels; however, after discontinuing the medication, cTnI levels increased again [13]. Previous studies have reported that serum cTnI concentrations are elevated in cats with HCM, which further increase during episodes of heart failure [14]. Additionally, cTnI levels in cats with HCM are associated with cardiac-related mortality and SAM [15,16].

In humans with HCM, LVOTO exceeding a moderate level (LV pressure gradient ≥ 50 mmHg) is both a therapeutic target and a recognized poor prognostic factor [17]. In contrast, LVOTO has not been shown to contribute to prognosis in cats with HCM [2], and the effects of LVOTO on myocardial injury remain unclear. Therefore, this study aimed to evaluate the relationship between myocardial injury and cardiac function based on LVOTO in cats with HCM.

## 2. Materials and Methods

This retrospective observational study was conducted in accordance with the Guidelines for Institutional Laboratory Animal Care and Use of our university. Additionally, the study was approved by the Etical Committee for Animal Use at our university’s medical teaching hospital (approval number: R2-4). Written informed consent for the use of patient evaluation data was already obtained from each cats’ owner.

### 2.1. Animals

A total of 46 client-owned cats diagnosed with HCM at our university’s medical teaching hospital between May 2021 and November 2024 were retrospectively included in the study. All cats underwent a complete physical examination, blood pressure measurement (using the oscillometric method), six-lead electrocardiography, and echocardiography. The diagnosis of HCM was based on maximal diastolic LV wall thickness of 6 mm or more, as determined by echocardiographic examination. Maximal LV wall thickness was obtained from the right parasternal long-axis or short-axis view, using the mean values of three consecutive cardiac cycles of the thickest segment. Based on the physical examination, blood pressure measurement, ultrasonography, and blood tests including thyroid hormone concentration, this study excluded cats with cardiac diseases other than HCM, systemic hypertension (systolic systemic blood pressure > 160 mmHg), hyperthyroidism, or other conditions that could cause myocardial hypertrophy other than HCM. No dehydration was observed in all cats. Cats that received the same cardiac medication for more than one month were included in this study. Only one cat diagnosed with HOCM has administered gabapentin (100 mg/head) one hour before examinations.

Cats diagnosed with HCM were classified based on American College of Veterinary Internal Medicine consensus guideline; Stage B1, B2, and C/D. Stage B1 was defined as asymptomatic cats with no or mild left atrial enlargement (left atrial-to-aortic ratio [LA/Ao] ≤ 1.6); stage B2 was defined as asymptomatic cats with moderate to severe left atrial enlargement (LA/Ao > 1.6); Stage C/D was defined as symptomatic cats with the evidence of current/previous congestive heart failure or atrial thromboembolism based on echocardiography and/or radiography [18,19]. Additionally, HCM cats with visually observed systolic anterior motion of the mitral valve based on B-mode images and a maximal LV outflow tract velocity (LVOTV) greater than 3.5 m/s on continuous wave Doppler imaging were classified as having HOCM. Cats not meeting these parameters were classified as having HNCM [2,20,21].

### 2.2. Standard Echocardiography

Conventional two-dimensional and Doppler ultrasonography were performed by a single investigator (R.S.) using an echocardiographic system equipped with a 9 MHz transducer (Vivid E95, GE Healthcare, Tokyo, Japan). During echocardiography, lead II electrocardiography was simultaneously recorded.

All data were obtained over three consecutive cardiac cycles. A single trained observer (H.K.) analyzed the images using an off-line workstation (Echo PAC PC version 201, GE Healthcare, Tokyo, Japan). The observer was not a member of the cardiology team that performed echocardiography and was blinded regarding the classification criteria of cats with HCM during the analysis. The LA/Ao was obtained from the B-mode image in the right parasternal short-axis view. The end-diastolic interventricular septal thickness (IVSd), end-diastolic LV posterior wall thickness (LVPWd), end-diastolic LV internal dimension (LVIDd), end-systolic LV internal dimension (LVIDs), and fractional shortening (FS) were obtained using B-mode in the right parasternal short-axis view and trailing edge-to-leading edge method. Measurements of IVSd and LVPWd were performed separately from maximal LV wall thickness [19]. Relative LV wall thickness (RWT) was also calculated using the following formula [22]:

RWT = (IVSd [mm] + LVPWd [mm])/LVIDd [mm](1)

Transmitral flow was evaluated from the left apical 4-chamber view, measuring the peak velocity of the early-diastolic wave (E wave) and peak velocity of the late-diastolic wave (A wave). The E wave to A wave velocity ratio (E/A) was also calculated; if E and A waves were fused, values for those cases were excluded from statistical analysis.

The LVOTV was measured using continuous wave Doppler in the left apical 5-chamber view. The minimum value at the lowest average heart rate during examination was defined as LVOTV_rest_, while the maximum value was defined as LVOTV_excited_. No special actions were taken to obtain LVOTV_rest_ and LVOTV_excited_.

### 2.3. Two-Dimensional Speckle Tracking Echocardiography

An overview of the 2D-STE analysis for cats has been previously described [4,6]. The analysis was performed using high quality images obtained from conventional echocardiography. For evaluating LV circumferential deformation, images of LV at the level of the papillary muscle were acquired in the right parasternal short-axis view. LV longitudinal deformation was evaluated using the left apical 4-chamber view. Longitudinal and circumferential strains (SL and SC, respectively) were obtained in the endocardium, epicardium, and whole layers of the LV. The endocardial to epicardial strain ratio (Endo/Epi), which may reflect compensatory mechanisms by endocardial layer, was also calculated [23,24]. The mean values of the measurements from three consecutive cardiac cycles obtained from high-quality images were used for all statistical analyses.

### 2.4. cTnI Measurement

Venous blood sampling was performed on the same day as echocardiography. Whole blood was promptly placed into a serum separation tube and centrifuged at 3000 rpm for 15 min at 4 °C. All serum samples were then stored at −80 °C. The chemiluminescence immunoassay method used to measure cTnI was outsourced (FUJIFILM VET Systems Co., Ltd., Tokyo, Japan). The reference range was set at 0.121 ng/mL or lower.

### 2.5. Statistical Analysis

Values obtained in this study were expressed as the median (interquartile range). All statistical analyses were performed using commercially available software for statistical analyses (EZR version 1.62) [25]. Categorical data were compared using Fisher’s exact test. The normality of continuous data was assessed using the Shapiro–Wilk test. For comparisons between HNCM and HOCM, normally distributed data were analyzed using Student’s t-test, while non-normally distributed data were analyzed using the Mann–Whitney U test. The relationship between echocardiographic variables and cTnI was assessed using Pearson’s correlation coefficient for normally distributed data and Spearman’s correlation coefficient for non-normally distributed data. Correlation analyses were conducted separately for HNCM and HOCM groups.

A *p*-value of <0.05 was considered statistically significant.

## 3. Results

### 3.1. Clinical Prifiles and Standard Echocardiography

**Figure 1:**
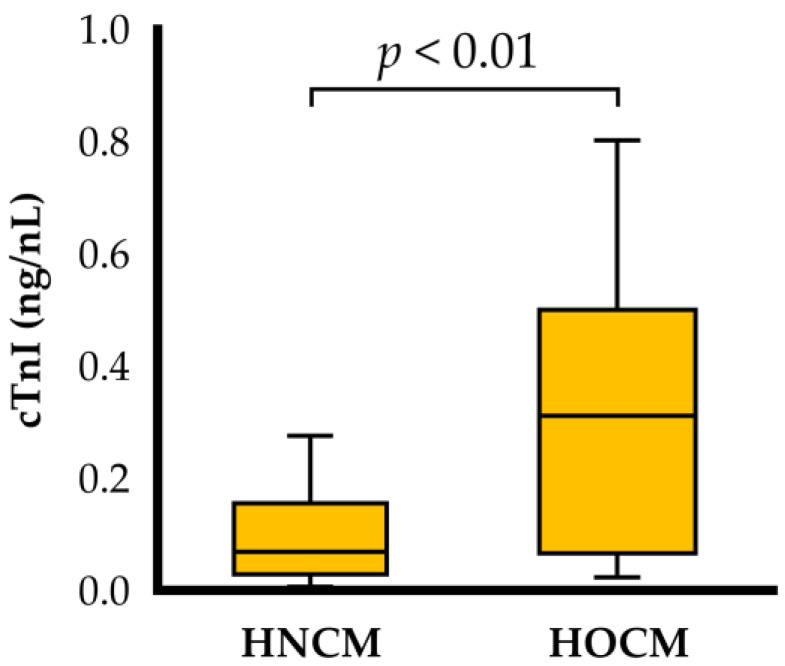
Box and whisker plots of cardiac troponin I in cats with obstructive and non-obstructive hypertrophic cardiomyopathy. Individual data points are shown. cTnI: cardiac troponin I; HNCM: non-obstructive hypertrophic cardiomyopathy; HOCM: obstructive hypertrophic cardiomyopathy.

The characteristic data for the cats included in the study with HNCM and HOCM are summarized in Table 1. This study consisted of 13 cats with HNCM and 33 cats with HOCM. Age, body weight, ACVIM staging, blood pressure, and heart rate did not differ significantly between the HNCM and HOCM groups. Medical therapy was given to 9 cats in the HNCM group and 13 cats in the HOCM group (Table 2). The cTnI concentration was significantly higher in the HOCM group compared to the HNCM group (*p* < 0.05, HOCM group: 0.311 [0.066, 0.500] ng/mL, HNCM group: 0.069 [0.029, 0.156] ng/mL, respectively). The cTnI levels were elevated in 31% of cases in the HNCM group and 73% of cases in the HOCM group (Figure 1).

Echocardiographic variables other than 2D-STE for cats with HNCM and HOCM are summarized in Table 3. All echocardiographic variables except for LA/Ao, did not differ significantly between HNCM and HOCM.

### 3.2. 2D-STE Variables

**Figure 2:**
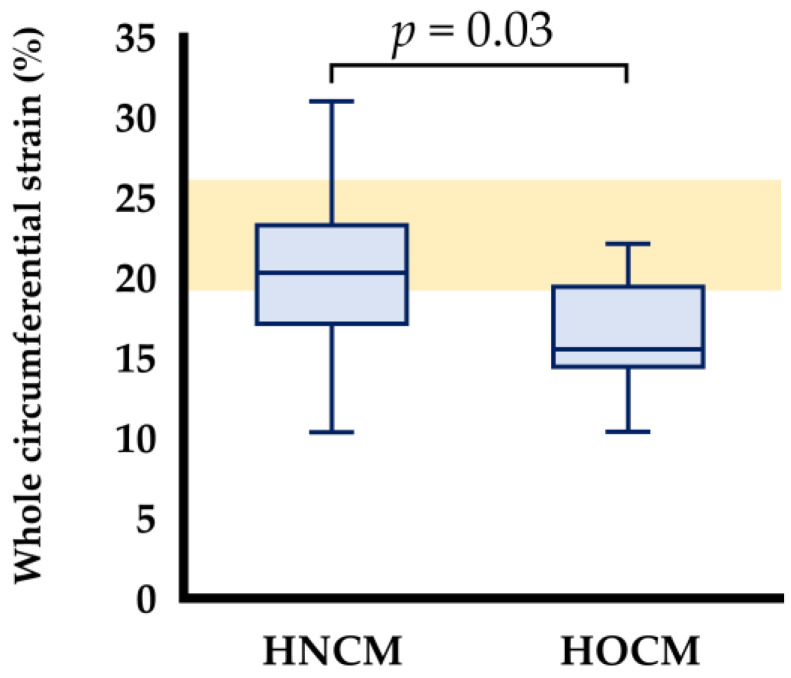
Box and whisker plots of circumferential strains in the whole layer in cats with obstructive and non-obstructive hypertrophic cardiomyopathy. Individual data points are shown. Orange bar shows the reference range of healthy cats (−19.3 to −26.0) noted in the previous study [9]. HNCM: non-obstructive hypertrophic cardiomyopathy; HOCM: obstructive hypertrophic cardiomyopathy.

The global 2D-STE data for cats with HNCM and HOCM are summarized in Table 4. The SC in the whole layer and epicardial layers was significantly lower in the HOCM group compared to the HNCM group (*p* < 0.05) (Figure 2). However, there was no significant difference between the HNCM group and the HOCM group in the SL of the whole and endocardial layers. Furthermore, the Endo/Epi of SL and SC also showed no significant differences between the two groups.

### 3.3. Correlation Analyses

Results of correlation analyses among echocardiographic indices in 33 cats with HOCM are summarized in Table 5. Significant positive correlations were found between cTnI concentration and LVOTV. However, there were no significant correlations between cTnI concentration and the SL, the SC. Additionally, significant correlations were not observed between the LVOTV and the SL, the SC.

In contrast, cTnI showed no significant correlations with LVOTV in 13 cats with HNCM (vs. LVOTV_excited_: *p* = 0.69; vs. LVOTV_rest_: *p* = 0.69). Additionally, there was no significant correlation between cTnI and myocardial strain (vs. whole SL: *p* = 0.48; vs. whole SC: *p* = 0.10). Furthermore, LVOTV and myocardial strain did not have significant correlations (LVOTV_excited_ vs. whole SL: *p* = 0.35; LVOTV_excited_ vs. whole SC: *p* = 0.45; LVOTV_rest_ vs. whole SL: *p* = 0.33; LVOTV_rest_ vs. whole SC: *p* = 0.65).

## 4. Discussion

This study investigated the association between LVOTO, LV myocardial function evaluated using 2D-STE, and myocardial injury based on cTnI in cats with HCM. Significantly worsened SC and higher cTnI were observed in cats with HOCM. These results of the present study indicated that LVOTO could cause myocardial injury and myocardial dysfunction.

In this study, cTnI levels were significantly higher in the HOCM group compared to HNCM group, which is consistent with previous studies [16]. Furthermore, cTnI levels showed a positive correlation with LVOTV. To the best of the authors’ knowledge, this is the first report of such findings in cats. Some previous studies showed that elevated cTnI levels were associated with cardiac-related mortality and disease progression in cats with HCM [14,15]. In human medicine, LV pressure overload caused by LVOTO is thought to induce myocardial hypertrophy, absolute and relative myocardial ischemia, and fibrosis, ultimately leading to myocardial injury and increased cTnI levels [13,26]. Furthermore, decrease in cTnI levels has been observed with the alleviation of LVOTV by medical therapy [13]. Hence, cTnI is used to monitor treatment effects in human HCM. Overall, our results suggest that LVOTO may exacerbate myocardial injury also in cats with HCM, as seen in humans. However, these findings were inconsistent with the previous report that cTnI was not associated with LVOTV in cats with SAM but without LV hypertrophy [27]. The differences in these results might be caused by the difference in study design and the labile nature of SAM and LVOTO [16]. Further studies including a larger study population are warranted to ensure the validity of our results.

The LV SC in the whole and epicardial layers were significantly lower in cats with HOCM. These findings were consistent with previous reports on human HCM and feline HOCM compared to healthy subjects, in which the reduction in strain of the whole and epicardial layers is considered to reflect myocardial dysfunction in HCM [3,5]. Because this study classified cats with HCM according to LVOTV and there was no significant difference in disease progression based on ACVIM stage, our results indicated that the presence of LVOTO might worsen myocardial function, especially in the circumferential direction, in cats with HOCM.

On the other hand, no significant difference between HOCM and HNCM group was observed in circumferential endocardial to epicardial strain ratio in this study, which is consistent with previous reports [16]. Circumferential endo/epi layer ratio is considered a compensatory response of endocardial layer strain to the reduction in epicardial layer strain [24]. Therefore, in HOCM without congestive heart failure, this compensatory mechanism is thought to be preserved.

The present study showed no significant correlation between cTnI levels and myocardial strain in cats with HOCM. We previously reported that feline HCM could decrease LV SL during the early stages of the disease compared to healthy cats, with further reductions as the disease progresses [4]. This finding is consistent with observations in humans with HCM, whereby pathological changes such as fibrosis could precede anatomical changes in myocardial hypertrophy [28]. Furthermore, the previous study reported that these pathological changes could be detected using SL analysis [28]. Therefore, the decrease in SL is likely indicative of a decline in myocardial function common to HCM regardless of the presence of LVOTO, which might explain the lack of correlation between SL and cTnI in HOCM. Additionally, a half-life period of cTnI might affect the results. Unfortunately, there are no reports regarding the half-life of cTnI in cats; however, the half-life of cTnI in humans and dogs are reported to be relatively short (2.0 h and 1.85 h, respectively) [29,30]. Although cTnI levels rise immediately after myocardial injury and reach a peak within a few hours, organized myocardial tissue through chronic myocardial remodeling might rather decrease the release of troponin from myocardium. Therefore, especially in HCM which shows chronic course of myocardial remodeling, cTnI might not be necessarily associated with myocardial function. However, the results of this study were based on a relatively small sample size, which may have limited statistical power. Future studies are expected to evaluate the association between myocardial function and cTnI before and after medical therapy for LVOTO in the same HOCM cases.

In humans, LVOTO is considered a poor prognostic factor and a target for medical intervention [17]. Pharmacological treatments include beta-blockers, calcium-channel blockers, disopyramide, and cardiac myosin inhibitors [31,32,33,34,35]. In some cases, surgical approaches may also be considered [36]. In contrast, a previous study on cats with HCM did not identify LVOTO as a prognostic factor [2]. As for the cause of this result, we consider that the relatively low LVOTO criteria of LVOTV > 2.5 m/s might have led to the classification of obstructive HCM cases that were not clinically problematic [2]. Whereas, in this study, LVOTV showed positive correlation with cTnI, but this finding was not observed in HNCM. A previous study reported that abnormally high levels of cTnI (> 0.7 ng/mL) were associated with poor prognosis in cats with HCM [15]. Therefore, our results suggest that excessively high LVOTV (i.e., severe LVOTO) may indirectly affect prognosis through elevated cTnI levels. In other words, severe LVOTO should be regarded as a target for therapeutic intervention. Future research is needed to investigate the relationship between the degree of LVOTO and prognosis in cats with HOCM.

In humans, even if the pressure gradient in the LV outflow tract is ≤ 30 mmHg at rest, evaluation using stress-induced LVOTV should be considered in determining therapeutic intervention since gradients could vary with heart rate, blood pressure, volume status, activity, medications, food, and alcohol intake [37]. Although there were no differences in our results between LVOTV_excited_ and LVOTV_rest_ in this study, LVOTO and SAM are labile in cats with HCM. Given that LVOTO is associated with increased cTnI and myocardial dysfunction, there is a potential benefit of assessing LVOTV at multiple conditions.

This study has several limitations. First, as a retrospective study, it included cases in which medications had been prescribed at the time of evaluation, as well as cases that had already started treatment at the time of first visit. This might have influenced the disease status of cardiomyopathy across groups. Second, the diagnosis of hypertrophic cardiomyopathy was based on echocardiographic findings, and pathological confirmation for diagnosing HCM and detecting myocardial degeneration was not available for all cases. Future studies should focus on intergroup comparisons (HNCM vs. HOCM) using cases prior to treatment, employing unified ACVIM staging.

## 5. Conclusions

In cats with HCM, LVOTO appears to have a close relationship with cTnI levels and myocardial function. Based on these findings, LVOTO in cats with HOCM may exacerbate LV myocardial injury and dysfunction; its potential as a target for medical treatment is indicated. Future studies with larger sample sizes are warranted to further investigate the relationship between LVOTO and long-term prognosis.

## Figures and Tables

**Table 1:** Clinical characteristics of cats with hypertrophic cardiomyopathy enrolled in this study.

Variables	HNCM (n = 13)	HOCM (n = 33)	*p*-Value
Sex (male, female)	6, 7	23, 10	0.18
Age (year)	3.4 (1.7, 7.1)	2.7 (1.4, 5.7)	0.45
Body weight (kg)	4.2 (3.6, 4.9)	4.3 (3.8, 5.4)	0.35
ACVIM (B1, B2, C/D)	12, 1, 0	30, 3, 0	0.99
Heart rate (bpm)	180 (132, 202)	192 (166, 229)	0.79
Systolic blood pressure (mmHg)	131 (125, 140)	135 (121, 142)	0.26
LVOTV_rest_ (m/s)	1.1 (0.9, 1.3)	3.7 (2.8, 4.5) *	<0.01
LVOTV_excited_ (m/s)	1.4 (1.2, 1.9)	4.3 (4.0, 4.9) *	<0.01
cTnI (ng/mL)	0.069 (0.029, 0.156)	0.311 (0.066, 0.500) *	<0.01
Number of cases with high cTnI levels (n)	4	24	0.02
Medication (yes, no)	9, 4	13, 20	0.10

*: The value was significantly different between HNCM and HOCM groups (*p* < 0.05). LVOTV: left ventricular outflow tract velocity, HNCM: non-obstructive hypertrophic cardiomyopathy, HOCM: obstructive hypertrophic cardiomyopathy.

**Table 2:** Medical treatment received by cats with hypertrophic cardiomyopathy.

Medical Drugs	HNCM (n = 13)	HOCM (n = 33)	*p*-Value
Beta blocker (n)	7	10	0.18
Angiotensin converting enzyme inhibitor (n)	1	2	0.99
Pimobendan (n)	1	1	0.99
Clopidogrel (n)	2	0	0.36
No medication (n)	4	20	0.10

LVOTV: left ventricular outflow tract velocity, HNCM; non-obstructive hypertrophic cardiomyopathy; HOCM: obstructive hypertrophic cardiomyopathy.

**Table 3:** Results of echocardiographic variables in cats with obstructive and non-obstructive hypertrophic cardiomyopathy.

Variables	HNCM	HOCM	*p*-Value
LA/Ao	1.2 (1.1, 1.2)	1.3 (1.2, 1.4) *	0.049
IVSd (mm)	6.0 (5.0, 7.1)	6.3 (5.3, 7.0)	0.52
LVPWd (mm)	5.2 (4.6, 6.6)	5.8 (5.4, 6.9)	0.053
LVIDd (mm)	13.3 (11.4, 15.5)	12.8 (11.9, 15.3)	0.85
RWT	0.90 (0.81, 0.93)	0.94 (0.76, 1.08)	0.28
FS (%)	42.2 (37.4, 54.9)	43.0 (37.9, 48.4)	0.92
E vel (m/s)	0.6 (0.5, 0.7)	0.8 (0.7, 0.9) *	0.01
E/A	0.8 (0.8, 1.1)	0.9 (0.8, 1.3)	0.45
E/A fusion	1.0 (0.8, 1.0) (n = 5)	1.0 (0.9, 1.1) (n = 9)	0.62

*: The value was significantly different between HNCM and HOCM groups (*p* < 0.05). A: late-diastolic transmitral flow velocity, E: early-diastolic transmitral flow velocity, FS: fractional shortening, IVSd: end-diastolic interventricular septum thickness, LA/Ao: left atrial to aortic diameter ratio, LVIDd: end-diastolic left ventricular internal dimension, LVPWd: end-diastolic left ventricular posterior wall thickness, HNCM: non-obstructive hypertrophic cardiomyopathy, HOCM: obstructive hypertrophic cardiomyopathy, RWT: relative wall thickness.

**Table 4:** Results of two-dimensional speckle tracking variables in cats with obstructive and non-obstructive cardiomyopathy.

	HNCM	HOCM	*p*-Value
Longitudinal strain (%)			
Whole layer	13.1 (10.9, 14.2)	13.0 (9.2, 16.9)	0.89
Endocardium	15.0 (12.6, 16.2)	14.0 (10.8, 20.1)	0.94
Epicardium	11.6 (9.4, 12.9)	10.7 (8.0, 13.9) *	0.52
End/Epi	1.3 (1.2, 1.4)	1.3 (1.2, 1.6)	0.70
Circumferential strains (%)			
Whole layer	20.3 (16.0, 23.5)	15.5 (14.3, 19.7) *	0.028
Endocardium	37.2 (31.8, 41.1)	31.8 (28.6, 35.6)	0.07
Epicardium	8.9 (7.3, 12.0)	6.6 (5.0, 8.8) *	0.011
End/Epi	4.2 (3.2, 5.3)	4.7 (3.8, 5.8)	0.23

*: The value was significantly different between HNCM and HOCM groups (*p* < 0.05). HNCM: non-obstructive hypertrophic cardiomyopathy; HOCM: obstructive hypertrophic cardiomyopathy.

**Table 5:** Results of correlation analysis among echocardiographic variables in 33 cats with HOCM.

	cTnI		LVOTV_excited_		LVOTV_rest_	
	*r*	*p*	*r*	*p*	*r*	*p*
cTnI	―	―	0.51	<0.01	0.50	<0.01
LVOTV_excited_			―	―	0.72	<0.01
LVOTV_rest_					―	―
Longitudinal strain (%)						
Whole layer	−0.27	0.13	−0.02	0.90	−0.01	0.98
Endocardium	−0.19	0.28	−0.09	0.64	−0.03	0.87
Epicardium	−0.29	0.10	−0.15	0.39	−0.15	0.26
Circumferential strains (%)						
Whole layer	−0.20	0.26	−0.08	0.67	−0.18	0.30
Endocardium	−0.13	0.46	−0.12	0.51	−0.07	0.71
Epicardium	−0.12	0.10	−0.03	0.85	−0.20	0.26

cTnI: cardiac troponin I; LVOTV: left ventricular outflow tract velocity.

## Data Availability

The datasets used or analyzed in the current study are available from the corresponding author upon reasonable request.
